# Non-topographical contrast enhancement in the olfactory bulb

**DOI:** 10.1186/1471-2202-7-7

**Published:** 2006-01-24

**Authors:** Thomas A Cleland, Praveen Sethupathy

**Affiliations:** 1 Department of Neurobiology and Behavior, Cornell University, Ithaca, NY 14853, USA

## Abstract

**Background:**

Contrast enhancement within primary stimulus representations is a common feature of sensory systems that regulates the discrimination of similar stimuli. Whereas most sensory stimulus features can be mapped onto one or two dimensions of quality or location (e.g., frequency or retinotopy), the analogous similarities among odor stimuli are distributed high-dimensionally, necessarily yielding a chemotopically fragmented map upon the surface of the olfactory bulb. While olfactory contrast enhancement has been attributed to decremental lateral inhibitory processes among olfactory bulb projection neurons modeled after those in the retina, the two-dimensional topology of this mechanism is intrinsically incapable of mediating effective contrast enhancement on such fragmented maps. Consequently, current theories are unable to explain the existence of olfactory contrast enhancement.

**Results:**

We describe a novel neural circuit mechanism, non-topographical contrast enhancement (NTCE), which enables contrast enhancement among high-dimensional odor representations exhibiting unpredictable patterns of similarity. The NTCE algorithm relies solely on local intraglomerular computations and broad feedback inhibition, and is consistent with known properties of the olfactory bulb input layer. Unlike mechanisms based upon lateral projections, NTCE does not require a built-in foreknowledge of the similarities in molecular receptive ranges expressed by different olfactory bulb glomeruli, and is independent of the physical location of glomeruli within the olfactory bulb.

**Conclusion:**

Non-topographical contrast enhancement demonstrates how intrinsically high-dimensional sensory data can be represented and processed within a physically two-dimensional neural cortex while retaining the capacity to represent stimulus similarity. In a biophysically constrained computational model of the olfactory bulb, NTCE successfully mediates contrast enhancement among odorant representations in the natural, high-dimensional similarity space defined by the olfactory receptor complement and underlies the concentration-independence of odor quality representations.

## Background

**Figure 1:**
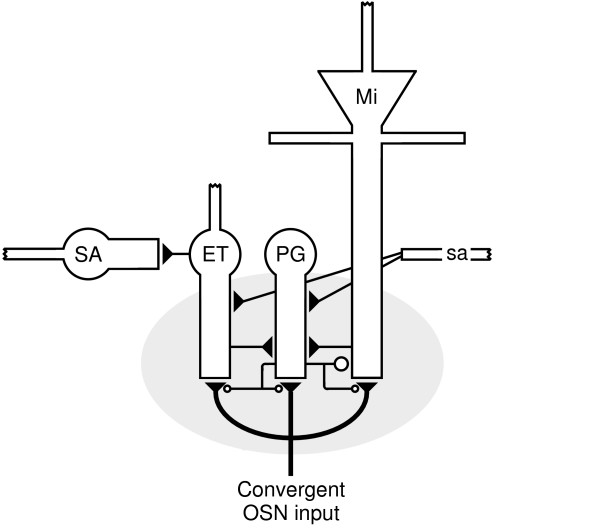
**Circuitry of the olfactory bulb glomerulus**. Primary olfactory sensory neurons (OSNs) that express a given odorant receptor and hence exhibit a common molecular receptive range converge upon discrete glomeruli in the olfactory bulb input layer. OSN terminal arborizations form excitatory synapses with the primary dendrites of mitral (Mi), periglomerular (PG) and external tufted (ET) cells; the latter in turn activate short-axon (SA) and local PG cells. PG cells in turn synapse reciprocally with local mitral cell primary dendrites; the mitral cells excite the PG dendrites while the latter inhibit mitral cell primary dendrites via graded inhibition. SA cells project axons (sa) broadly into other glomeruli [2], within which they excite ET and PG cells; in the present model, the resulting lateral excitatory network of ET and SA cells (the ET/SA network) provides normalizing feedback inhibition to mitral cells via their excitation of PG cells. Mitral cells are the primary output neuron of the olfactory bulb, projecting via axon collaterals to several cortical and subcortical target regions [4]. *Filled triangles *denote excitatory synapses; *open circles *denote inhibitory synapses. *Lower case labels *denote incoming processes originating in other glomeruli. Adapted from reference [2].

Primary olfactory sensory neurons (OSNs) line the nasal epithelium and respond to the presence of odors that diffuse through the nasal mucus layer and bind to olfactory receptors expressed on OSN cilia. Each OSN expresses only one or a few species of olfactory receptor, which define the molecular receptive range [1], or chemical receptive field, of that OSN. The axons of several thousand OSNs expressing the same receptors converge onto a few discrete glomeruli in the olfactory bulb, within which they form glutamatergic synapses with the dendrites of mitral cells, periglomerular cells, and external tufted cells. Both mitral and external tufted cells also form excitatory synapses onto periglomerular cells within the same glomerulus, whereas the periglomerular cells inhibit local mitral cells as well as presynaptically inhibiting OSN output synapses [2,3] (Figure 1). The mitral cells (along with middle and deep tufted cells) are the primary output neurons of the olfactory bulb, projecting via axon collaterals to multiple central structures within the brain [4]. A detailed description of these and other synaptic relationships within the olfactory bulb has been provided by Shipley *et al. *[5].

Due to the convergence of OSNs expressing the same olfactory receptor proteins, the pattern of activated glomeruli on the surface of the olfactory bulb reflects the pattern of activated olfactory receptors, identifying the constellation of chemical qualities that together constitute the presented odor. Many studies have attempted to elucidate the organization of this chemosensory map on the surface of the olfactory bulb. However, while great efforts have been made to infer meaningful patterns in these data, essentially all such studies have revealed fragmented, patchy maps without a clear, predictive ordering of the characteristics of chemical stimuli. This result stands in sharp contrast to analogous sensory maps in, for example, somatosensory, visual, and auditory cortices, in which neurons with overlapping receptive fields are located in predictable locations adjacent to one another [6]. Fragmented stimulus quality maps such as those observed in the olfactory bulb pose unique problems for stimulus processing.

Contrast enhancement is a common property of sensory systems that serves to narrow, or sharpen, sensory representations by specifically inhibiting neurons on the periphery of the representation – e.g., the edges of a retinal image – in order to enhance its figure-background contrast. In many sensory systems, contrast enhancement is mediated by lateral inhibitory projections. Crucially, the effectiveness of lateral inhibition in this context depends upon the topographical mapping of stimulus similarities within the relevant brain region, such that the physical proximity of neurons reliably reflects the similarity of the information that they mediate. In the retina, for example, the spatial contrast of visual images is enhanced by lateral inhibitory projections within the two dimensions of the retinal field [7]. Overlapping regions of the visual field are sampled by adjacent photoreceptors; hence, physically neighboring neurons mediate similar sensory information. Consequently, the projection of inhibition by neurons onto their physical neighbors is an effective means of projecting inhibition onto those neurons that mediate similar sensory information. In the auditory system, frequency tuning in the inferior colliculus and medial geniculate body is similarly sharpened along the single dimension of frequency [8,9]. One-dimensional frequency tuning maps in auditory structures ensure that physically neighboring neurons will encode similar sound frequencies; hence, nearest-neighbor lateral inhibition again can effect mutual inhibition among similarly-tuned neurons. Critically, this mechanism of contrast enhancement is effective in these two modalities only because they are both low-dimensional. As neural cortices are layered structures, and thus functionally two-dimensional, contrast enhancement via nearest-neighbor lateral inhibition is effective only for modalities in which the relevant similarities among stimulus elements can be mapped continuously onto two or fewer dimensions.

While odor quality representations within the olfactory bulb are clearly sharpened by contrast enhancement [10], the fragmentation of odor maps indicates that the physical proximity of glomeruli cannot reliably connote similarity in their molecular receptive ranges, rendering nearest-neighbor lateral inhibition ineffective at sharpening odor stimulus representations (see below). Indeed, quantitative analyses of calcium imaging data from the analogous insect antennal lobe have demonstrated that glomerular tuning similarities do not correlate with their physical proximity, and that a pattern of functional inhibition reflecting the former, not the latter, better reproduces the observed input-output function of antennal lobe glomeruli [11]. While such a proximity-independent functional topology is theoretically achievable using targeted lateral projections (i.e., selective inhibitory projections to similarly-tuned glomeruli irrespective of physical proximity), the combinatorial requirements for the formation of such a projection pattern are intractable [12,13]. Furthermore, the probability of receptive field overlap between odor receptors is not only a function of these receptors' structures, but also of the statistics of the chemical environment. As the distribution of odor stimuli in natural environments is constantly in flux, quantitative measures of tuning similarity among glomeruli (and hence the appropriate target glomeruli for lateral inhibition) will not remain stable over time. Hence, any contrast enhancement mechanisms dependent upon specific morphological projections will be poorly optimized for most odor environments. An alternative neural circuit algorithm is required, one that can both perform similarity-dependent computations on fragmented representations and continually remain optimized with respect to changing chemical environments.

We here propose and demonstrate a novel neural mechanism, non-topographical contrast enhancement (NTCE), which permits the regulation of olfactory tuning curves in an arbitrarily high-dimensional sensory space, using established olfactory bulb circuitry, without reliance on specific connections among glomeruli. This lack of dependence on the anatomical proximity of glomeruli enables the bulb to remain optimized with respect to the statistics of changing odor environments, and additionally renders the system robust to the generation of novel glomeruli over the course of development, evolution, or experimental manipulation [14-18]. The NTCE algorithm is consistent with, and in some cases explains, diverse physiological datasets from the olfactory bulb, and in particular replicates the canonical contrast enhancement results of Yokoi and colleagues [10]. We first present the core principles of NTCE in an illustrative model, and then demonstrate that it can be implemented by established glomerular layer circuitry using a biophysically constrained compartmental model of the olfactory bulb glomerular layer.

## Results and discussion

### Fragmented maps and the dimensionality of stimulus representations

All auditory stimuli can be mapped as spectra along a single axis of frequency. Retinotopic images of visual stimuli can be unambiguously represented as two-dimensional patterns of ganglion cell activation. However, substantially higher-dimensional spaces are required to map the analogous sensory space in which odor stimuli are distributed [11,19-21]. Heuristically, this reflects the large number of different ways that one can gradually alter the molecular structure and charge distributions of odorous molecules or the component ratios of multicomponent odors; more concretely, it reflects the large number of independent sensors (i.e., types of odorant receptor) that provide the raw material for olfactory representations. Each glomerulus represents a convergent population of olfactory sensory neurons expressing the same odorant receptor protein, which determines its molecular receptive range; due to this convergence, glomeruli can be treated as selective, low-noise chemosensory units [22]. In mice, there are roughly 1000 such receptor types and hence roughly 1000 chemoreceptively distinct sets of glomeruli [23-25]. Odorant stimulation activates characteristic, odor-specific groups of glomeruli, and perceptual similarities in odor quality correlate with the similarities in these odor-evoked patterns of glomerular activation [26,27]. As each glomerulus is capable of being independently activated to differing degrees, whether by natural or artificial stimuli [28], there are roughly 1000 potential dimensions in mice along which odor quality representations may be gradually varied. Consequently, in order for nearest-neighbor relationships to reliably reflect odorant similarity in mice, a topographical map of odor representations would need to occupy an approximately 1000-dimensional similarity space. In layered neural structures, however, only two physical dimensions are available for topographical mapping.

**Figure 2:**
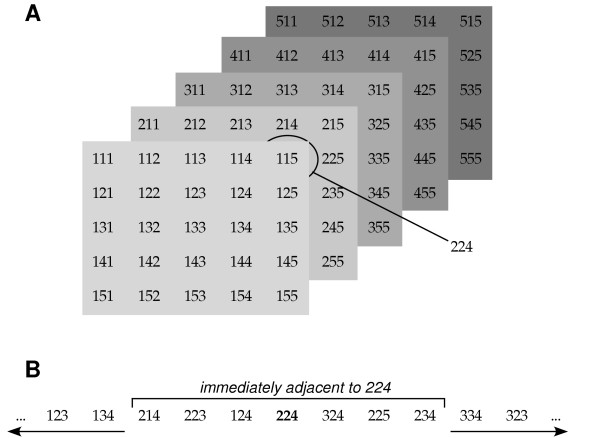
**Illustration of how the projection of high-dimensional odor representations onto lower-dimensional surfaces yields fragmented maps. A. **Three-dimensional matrix of numbers representing stimulus qualities, where each element is similar to its neighbors in proportion to their Euclidean distance. For clarity, each digit identifies the location of an element in one of these dimensions; hence, the element *333 *is at the center of the 5 × 5 × 5 cube depicted. **B. **The same three-dimensional matrix as in A, projected onto one dimension and only partially depicted. The six nearest neighbors (distance = 1 in Figure 2A) of element *224*, highlighted in *A*, are now clustered at distances of 1 to 3 from its location, retaining their nearest-neighbor relationships to the maximum extent possible. Consequently, the five remaining nearest neighbors of element *324 *(for example) can at best be clustered at distances of 3 to 7 from *324*, with the interposition of non-neighboring elements *225 *and *234*; subsequently, the four other nearest neighbors of element *225 *can at best be clustered at distances of 7 to 10 from its location, with an increasing number of non-neighboring interpositions (namely, the nearest neighbors of element *324*). Various optimized retentions of local similarity relationships can be obtained using a self-organizing map algorithm [29, 30] to generate a fragmented remapping of the three-dimensional matrix onto one dimension, but the fundamental problem is unavoidable: the distance relationships among elements in 3-space (Figure 2A) cannot be replicated in 1-space (Figure 2B).

As the olfactory sensory neuron complement converges onto the glomerular layer of the olfactory bulb, these high-dimensional representations of odorant similarity are projected onto the two physical dimensions of the olfactory bulb input layer, a process facilitated by the glomerular architecture of that layer. Mathematically, whenever high-dimensional representational patterns are projected onto lower-dimensional spaces, they form discrete maps, that is, patchy maps with embedded discontinuities (Figure 2). Neural network algorithms such as the self-organizing map [29,30], which perform such transformations adaptively, are able to retain local similarity relationships up to certain limits imposed by the topology of projection, at which point discontinuities are inevitable. One familiar example of this phenomenon in the nervous system is the Penfield-Rasmussen somatosensory homunculus [31,32], essentially a one-dimensional representation of the two-dimensional external body surface mapped coronally along the posterior parietal cortex. While body surface contiguity is maintained within discrete segments of this map, there are also sharp discontinuities mandated by its projection onto a one-dimensional surface as well as the presence of anisotropic elaborations (such as limbs) that disrupt contiguity relationships. For example, *thumb *and *neck *are represented in adjacent cortical regions, as are *toes *and *genitalia*, along with many more subtle examples, while representations of the arms and hands are interposed between those of the face and the rest of the head. The existence of such discontinuities in the neural representation is inevitable, as illustrated in Figure 2; furthermore, the frequency of the discontinuities will increase along with the difference between the intrinsic dimensionality of the representation and that of the neural substrate. Consequently, as the high-dimensional odor space defined by an animal's olfactory receptor complement and current chemical environment projects onto the two physical dimensions of the olfactory bulb glomerular layer, the chemotopic similarity map expressed thereon will be unavoidably fragmented, incorporating many embedded discontinuities. This is exactly what has been observed in numerous studies of odor representations across the glomerular layer of the olfactory bulb, although these commonalities have not always been emphasized by the different laboratories authoring these studies.

Substantial efforts have been made by several laboratories to elucidate an underlying order to the distribution of glomeruli across the surface of the olfactory bulb. While the multiple glomeruli activated by a given odorant stimulus are typically scattered, imaging studies of odorant-specific glomerular activation patterns have observed a broad tendency for the centroids of these activated groups to migrate to progressively more ventral regions as the odorant molecules become larger – e.g., straight-chain aldehydes of increasing aliphatic chain lengths – and more hydrophobic [33-35]. This phenomenon has been attributed to the differential sorption of odorants along the nasal inspiratory path (reviewed in [36]). Similarly, within the dorsal region of the bulb (the aspect most accessible to *in vivo *imaging studies), longer chain lengths have been associated with centroids of activation positioned more rostrally and laterally [34,37-39], though concentration artifacts may have influenced this finding [37]. Further studies have established *ad hoc *modules or clusters within the olfactory bulb that exhibit greater densities of glomeruli sensitive to particular odorant moieties than would be expected by chance [40-44]. However, these broad chemotopic profiles across the bulbar surface must not be confused with a reliable topography of feature similarity across the olfactory bulb. The chemotopic maps that have been described do not extend to the finer scale of individual glomeruli upon which contrast enhancement mechanisms operate [37,45]. In each of the imaging studies cited above, many glomeruli responding to similar odorant features are located at considerable distances from one another, and there are innumerable examples of strongly activated glomeruli located adjacent to unresponsive glomeruli in response to any given odorant – a pattern indicative of a highly fragmented map of odor quality. While both of these phenomena have been consistently observed in bulbar imaging studies, they have rarely attracted substantial discussion. The important consequence of these data is that, while some neighboring glomeruli are similarly tuned for odorant stimuli, others are not; hence, the physical proximity of glomeruli is not a reliable indicator of similarity in their molecular receptive ranges. For center-surround lateral inhibition to reliably mediate contrast enhancement, each of the odorant stimuli that substantially activate any given glomerulus must also activate all of its physical neighbors, albeit to a greater or lesser degree. This is not the case in the olfactory bulb.

### Glomerular circuitry implements non-topographical contrast enhancement (NTCE)

**Figure 3:**
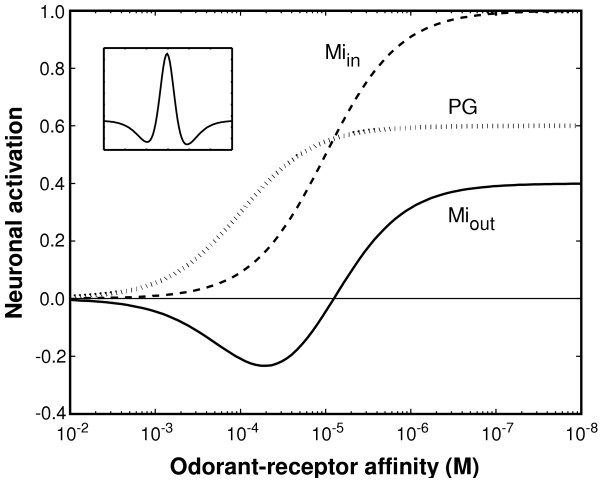
**The NTCE half-hat function depicted as the difference of two sigmoids**. A principal neuron (mitral cell Mi_in_, *dashed line*) and a local inhibitory interneuron (periglomerular cell PG, *dotted line*) are both directly, sigmoidally activated by increasing input levels (*abscissa*; here depicted as odorant-receptor affinity and neglecting odorant concentration). The local interneuron exhibits greater sensitivity to this input (i.e., it is half-activated by a weaker degree of odorant-receptor affinity) while the principal neuron has a greater maximum output amplitude. While input levels in a chemical binding context can conflate ligand-receptor affinity and ligand concentration [116], this ambiguity can be resolved by global feedback mechanisms (see text). When the two neurons are driven by the same input and the local interneuron inhibits the principal neuron, the net output activity of the principal neuron can become nonmonotonic with respect to input level, exhibiting a half-hat function capable of mediating contrast enhancement (mitral cell M_out_, *solid line*; see Methods). That is, with respect to the molecular receptive range of any glomerulus, the mitral cell output profile after NTCE (Mi_out_) will exhibit a narrower selectivity for odorants than do its associated olfactory receptors (Mi_in_). ***Inset***: Traditional one-dimensional "Mexican hat" contrast enhancement function, typically modeled as the difference of two Gaussians or as the difference between two differences of sigmoids (see Methods).

Contrast enhancement can be functionally defined as a process of competition between neurons proportional to the similarity of the information that they mediate; this definition is, of course, agnostic to mechanism. The olfactory bulb input layer provides precisely the architecture necessary to implement a form of non-topographical contrast enhancement that is not limited by its anatomically two-dimensional architecture. The NTCE algorithm described here depends upon two interacting computational mechanisms derived from established bulbar circuitry. The first mechanism operates independently within each glomerulus at the synaptic triad between OSN axons, periglomerular cell spines, and mitral cell apical dendrites [46-48]. Direct sensory input from OSN axonal arborizations activates both periglomerular neurons and mitral cell primary dendrites, while the periglomerular cells in turn inhibit local mitral cell primary dendrites via dendrodendritic synapses (Figure 1). This architecture creates a contrast enhancement generator element within each glomerulus that transforms mitral cell activity along a half-hat function (Equation 2, Figure 3; *see below*) such that mitral cells connected to moderately activated glomeruli – i.e., the "edges" of the high-dimensional odor representation – are specifically inhibited out of the active ensemble. When all glomeruli are combined, their collective output activity reflects the input pattern of glomerular activation filtered through a Mexican hat function with a maximum dimensionality equal to the number of glomeruli and a topology of similarity inherited from the odor environment. In other words, NTCE generates a pattern of inhibition equivalent to that which would be generated by a competitive lateral inhibitory network in which the strength of inhibition between any two glomeruli was proportional to the similarity in their molecular receptive ranges. This mechanism is illustrated below in an abstract model and in a uniglomerular compartmental model.

The second mechanism of NTCE is a global negative feedback loop utilizing the lateral excitatory network of external tufted and short-axon cells (the ET/SA network; Figure 1). This network feeds the average level of bulbar activity back upon all mitral cells as inhibition, which is necessary in order to moderate the influence of odor concentration and maintain the first NTCE mechanism within its effective dynamic range. This mechanism is illustrated below in a multiglomerular compartmental model.

### Computational principles: illustrative model

Irrespective of modality or dimensionality, successful contrast enhancement implies that the single or few most strongly activated units will yield robust output in response to a given stimulus, while output activity in more modestly activated units will be specifically inhibited and minimally-activated units will remain inactive. That is, output activity as a function of input activity yields a half-hat function (Figure 3, *Mi*_*out*_; see *Methods*), the generator of the familiar Mexican hat function (Figure 3, *inset*) and a signature function of contrast enhancement. As shown in a simple illustrative model (see Methods), this half-hat function can be generated independently within each glomerulus by driving parallel sigmoidal excitatory and inhibitory processes with the same sensory input, given that the inhibitory process both is more sensitive to that input than the parallel excitatory process and saturates at a lower activity level. Both of these conditions are favored by the much smaller volume and higher input resistance of periglomerular cell spines compared with mitral cell dendrites. Under these conditions, each glomerulus inhibits its own mitral cell-mediated output in scaled proportion to its sensory input, such that its net mitral output level exhibits a half-hat function along the axis of ligand-receptor affinity (Figure 3, *Mi*_*out*_). Hence, mitral cells innervating glomeruli that are the most strongly tuned to a given odorant feature will be activated, while mitral cells innervating moderately well-tuned glomeruli will be inhibited. Because of this natural attunement to relative degrees of glomerular activation irrespective of their location or interconnectivity, NTCE naturally inherits the intrinsic topology of the external chemosensory environment (as filtered by the animal's complement of primary odorant receptors). This is a critical property for a system likely to encounter environments in which the distribution of relevant odorants is unpredictable.

### Uniglomerular compartmental model

In order to determine whether the established electrophysiological and cytoarchitectonic properties of olfactory glomerular circuitry could indeed mediate these computations, we constructed a compartmental model of olfactory glomerular circuitry using the simulation language NEURON [49-51]. We first implemented a uniglomerular model in order to demonstrate the first of the two mechanisms underlying NTCE. The model included representations of mitral cells [52,53], periglomerular cells constrained to available anatomical and biophysical data and exhibiting appropriate single-cell physiological responses to inputs [2,47,54-58], and olfactory sensory neuron terminals designed to convey the average activation level of a coactivated and highly convergent olfactory sensory neuron population. To better illustrate the NTCE phenomenon, we limited the number of unconstrained parameters in our model and entirely omitted post-glomerular circuitry such as granule cells and mitral-granule reciprocal connectivity. Accordingly, we made no effort to model mitral cell bistability or complex spike patterning; the temporal window of interest in this model is the period preceding the first mitral cell spike, determining whether and when a mitral cell will first fire and hence preceding the recruitment of granule cells into the olfactory response. Mitral cell spike rates are used herein solely as a convenient measure of the degree of neural activation.

**Figure 4:**
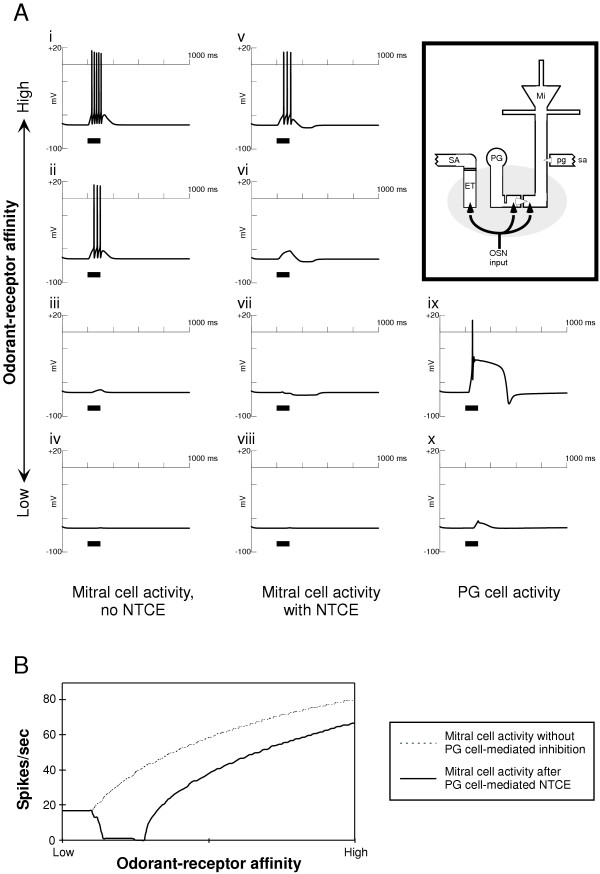
**Contrast enhancement generator in a single olfactory bulb glomerulus. Ai–Aiv**. Odor-evoked activity in model mitral cells as a function of odor ligand-receptor affinity, in the absence of periglomerular inhibition and neglecting stimulus concentration. Increasing odor ligand-receptor affinity generates a monotonic increase in mitral cell activation. **Av–Aviii. **The addition of periglomerular inhibition upon local mitral cells creates a contrast enhancement generator element by first inhibiting (panel *vii*), and then exciting (panels *vi, v*), mitral cells as odor ligand-receptor affinity increases. Inhibition was held constant, and panels *v–viii *depict the same four odor ligand-receptor affinities as are shown in panels *i–iv*. **Aix-Ax. **Periglomerular cell activation by the two lower-affinity odorant stimuli. While current input to periglomerular and mitral cells is identical, the greater input resistance and smaller volume of PG spines compared to mitral dendrites result in a greater voltage deflection in and hence a greater activation of PG cells (compare panels *iv *and *x*). Additionally, low-threshold T-type calcium current [55] evokes a near-maximal burst response from PG cells even at low input levels (panel *ix*), which mediates the mitral cell inhibition shown in panel *vii*. **B. **Mitral cell spike count over a 1 sec stimulus in the absence and presence of PG cell-mediated NTCE. In order to illustrate the effects of mitral cell inhibition, a 150 pA depolarizing current was continuously injected into the mitral cell soma to elicit a baseline spike rate. Mitral cell spiking is employed solely as an index of mitral cell activation; the present mitral cell model does not include complex spike patterning mechanisms. With intact NTCE, as odor ligand-receptor affinity increases, the mitral cell activation level reflects a half-hat function (Figure 3, *Mi*_*out*_). ***Inset***. Model architecture. OSN synaptic input activates mitral cell distal dendrite, periglomerular dendritic spine, and a combined ET-SA-PG function that projects inhibition onto all mitral cells other than that in the same glomerulus (*shaded regio*n). Synaptic weights were the same across all glomeruli, while odorant-receptor affinities differed. *Filled triangles*: excitatory synapses. *Open triangles*: inhibitory synapses. *Lower case labels *denote incoming processes originating in other glomeruli.

First, identical levels of olfactory sensory input were delivered to periglomerular dendritic spines and mitral distal dendritic tufts via glutamatergic synapses in a uniglomerular version of the model. We did not alter the relative sensitivities of the two neuron types to sensory input other than that afforded by the periglomerular spine's greater input resistance and smaller volume, though such a sensitivity difference could have been readily generated by manipulating postsynaptic receptor densities. The periglomerular cell spine also inhibited the mitral cell dendrite via a thresholded, graded mechanism. Stimulation of the OSN terminals of this network with low-affinity odorant stimuli generated no activity in mitral cells (Figure 4Aviii), while the mitral cell activation evoked by medium-affinity odorants was dissipated by periglomerular inhibition before spike initiation (Figure 4Avi–vii). Stimulation with the highest-affinity odorants, however, evoked activity in mitral cells that overcame PG-mediated inhibition and produced action potentials (Figure 4Av). Plotting mitral cell activation as a function of odor ligand-receptor affinity generated a half-hat function (Figure 4B) corresponding to that in the illustrative model (Figure 3, *Mi*_*out*_), demonstrating a successful, classical contrast enhancement generator element within a single glomerulus. In contrast, mitral cells exhibited monotonically increasing activation in response to presentation of higher-affinity odorants when PG-mediated inhibition was absent (Figure 4Aiv–i).

In our analysis, the established biophysical characteristics of glomerular neurons and synapses materially contribute to the effectiveness of NTCE. The synaptic triad between OSNs, periglomerular cell spines, and mitral cells [46-48] is optimal for enabling periglomerular inhibition to effectively shunt the synaptic excitation of mitral cells, owing to the close proximity of excitatory and inhibitory inputs [59-61]. The high input resistance, thin dendritic processes, and tiny spines of PG neurons effect a a a rapid local depolarization of membrane upon excitation by sensory input (Figure 4Aix–x). This enables the fast release of GABA onto mitral cell primary dendrites before the concomitant excitation of these larger, leakier dendrites can evoke an action potential in the electrotonically distant soma or somewhat nearer primary dendrite (Figure 4Aiii–iv; [52,53,62]. Indeed, recordings from mitral cells *in vivo *have shown fast inhibitory postsynaptic potentials (ipsps) preceding mitral cell spike initiation even in the most rapidly-responding mitral cells [58,63,64]. While this chloride-mediated shunt inhibition may often be insufficiently hyperpolarizing to evoke clearly visible ipsps, it is highly effective at blocking spike initiation by multiplicatively decrementing the cumulative effect of excitatory sensory input (Figure 4Avi–vii;[61,65]; see also [66]). Furthermore, the activation of low-threshold T-type calcium currents in PG cells [55], while not essential to contrast enhancement, enhances its efficacy by potentiating the effect of small inputs on local PG inhibitory output (Figure 4Aix). Finally, preintegrative mechanisms such as NTCE, in which inhibition is directed to mitral cell inputs prior to their integration into a spiking response, enjoy theoretical computational advantages that postintegrative lateral inhibitory mechanisms (such as those mediated by mitral-granule interactions) cannot provide, including an improved capacity to represent arbitrary combinations of overlapping inputs and to recognize partial input patterns [67,68].

### Multiglomerular compartmental model

**Figure 5:**
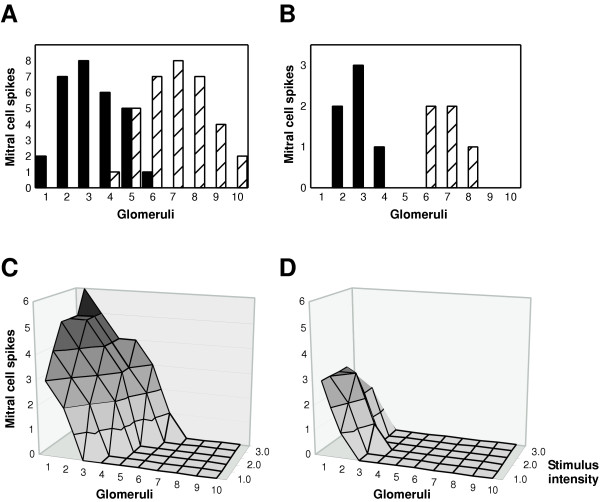
**Non-topographical contrast enhancement in a ten-glomerulus model. A. **Odor-evoked spike counts in mitral cells associated with ten different glomeruli exhibiting different affinities for odor ligands, in the absence of contrast enhancement processes. Response profiles for two similar odors are shown (*solid *and *striped *bars). For visual clarity, glomerular responses are ordered in this figure according to their affinities for the two model odorants used. **B. **Mitral cell responses to each of the same two odorants with NTCE processes intact. The addition of intraglomerular inhibition mediated by PG cells and interglomerular inhibition mediated by the ET/SA network strongly disambiguated mitral cell response profiles, abolishing the overlap between the two odorant representations. **C. **With intraglomerular periglomerular inhibition intact, but in the absence of interglomerular inhibition, mitral cell odorant representations broaden and spike counts rise as stimulus intensity increases. **D. **The addition of interglomerular feedback inhibition via the ET/SA network normalizes mitral cell activation patterns with respect to odorant concentration, resolving the potential ambiguity between ligand-receptor affinity and ligand concentration. Increased stimulus intensities neither dramatically alter the population of activated mitral cells nor increase spike rates in activated mitral cells. All odor stimuli in this figure were presented for 100 ms.

The second mechanism required by the NTCE algorithm is essentially a global inhibitory feedback loop. This mechanism, based on the ET/SA lateral excitatory network and inhibitory PG cells (Figure 1), mitigates the effects of absolute odor concentration, thus enabling mitral cell activity patterns to better reflect *relative *odor ligand-receptor affinities and efficacies and hence preserve the representation of odor quality across a reasonable concentration range. We constructed a multiglomerular version of this model in order to directly illustrate non-topographical contrast enhancement among multiple glomeruli differentially activated by the same odorant, and to assess the ability of the olfactory bulb ET/SA network to normalize bulbar responses to different odor concentrations via global feedback inhibition (see Methods). In the present model, ET/SA network activity was simulated by projecting slow synaptic inhibition from each of the other glomeruli onto mitral cell primary dendrites (Figure 4, *inset*), effectively translating the NTCE half-hat function of each glomerulus on its abscissa in proportion to average bulbar input intensity such that only a small and consistent population of the most strongly activated mitral cells overcame this activity-dependent inhibition. Ten glomeruli were interconnected via the ET/SA network such that all mitral cells received identically-weighted inhibitory inputs from each of the other nine glomeruli. The ten glomeruli were assigned different affinities for each test odorant such that each odorant representation constituted a unique ten-dimensional vector. Addition of intraglomerular inhibition mediated by periglomerular cells and global normalizing inhibition mediated by the ET/SA network strongly disambiguated the mitral cell response profiles evoked by two similar odorants (Figure 5A,B).

### Mechanisms of concentration compensation

The persistence of odor quality across concentrations is a difficult and continuing problem in olfaction. The most plausible explanation for this property is that common elements are retained among the representations of different concentrations of the same odorant, suggesting that some sort of normalization for concentration is likely to occur within the olfactory bulb. Indeed, such a normalization process has been proposed for the olfactory bulb based on imaging data showing that normalized odor-specific glomerular activity maps are relatively similar across stimulus concentrations [69,70], and that the degree of similarity in these normalized maps predicts similarities in perceived odor quality across concentrations more reliably than do non-normalized maps [69]. Consistent with these proposals, the NTCE circuit mechanism inherently compensates for concentration as the primary glomerular representation, measured predominantly from OSN presynaptic arbors, is transformed into the secondary representation based on patterns of activated mitral cells. Specifically, global feedback inhibition mediated via the ET/SA network serves to improve stability in odor quality representations among mitral cells across reasonable concentration ranges. When a given model odorant was applied at a series of concentrations (with synaptic weights unchanged), the higher concentrations recruited increasingly broad distributions of (presynaptically) activated glomeruli, as has been observed in imaging studies [38,69,71]. With intraglomerular inhibition intact, but in the absence of interglomerular inhibition, mitral cell activation patterns also broadened with increased odorant concentrations, as these higher concentrations recruited more weakly-tuned glomeruli into the ensemble of excited mitral cells (Figure 5C). However, with an intact interglomerular ET/SA network, each odorant concentration in the series could evoke comparable levels of mitral cell activity in a consistent, odor-specific subpopulation of mitral cells, conferring a degree of concentration-independence upon odor-specific mitral cell response profiles (Figure 5D). While the synaptic strengths underlying interglomerular inhibition could be tuned so as to produce either gradual recruitment or gradual elimination of mitral cells from the ensemble as odor concentration increased, a relative constancy in the ensemble of activated mitral cells is conservative in that odor-evoked mitral cell spike counts do not vary consistently with changing odorant concentrations [72-74], and certainly do not scale with the monotonically increasing presynaptic activation of olfactory glomeruli as shown in imaging studies. More specifically, given realistic odors that present multiple, diverse ligands for olfactory receptors, this constancy is only an average; individual mitral cells' responses would be expected to change qualitatively as their relative degrees of input activation change with respect to the population average. Indeed, mitral cell response profiles measured across concentration series have presented appropriately complex results; for example, nonresponsive mitral cells often become inhibited as odor concentration increases [72-74], while other mitral cells change from a net inhibitory response to a net excitatory response [73].

The normalization of olfactory representations for concentration does not imply that concentration information is lost. Model mitral cells excited by an odor stimulus exhibited shorter spike latencies as concentration increased (Figure 4), consistent with experimental data [63,75]. Concentration may also be represented in the degree of stimulus-evoked spike synchronization among mitral cells, as has been suggested for the insect antennal lobe [76]. Finally, middle and deep tufted cells, as well as the trigeminal system, may contribute to the representation and perception of odor intensity [77]. NTCE does not eliminate concentration information, but simply mitigates its impact on odor quality representations.

### Contrast enhancement tested with systematically varying odor stimuli

**Figure 6:**
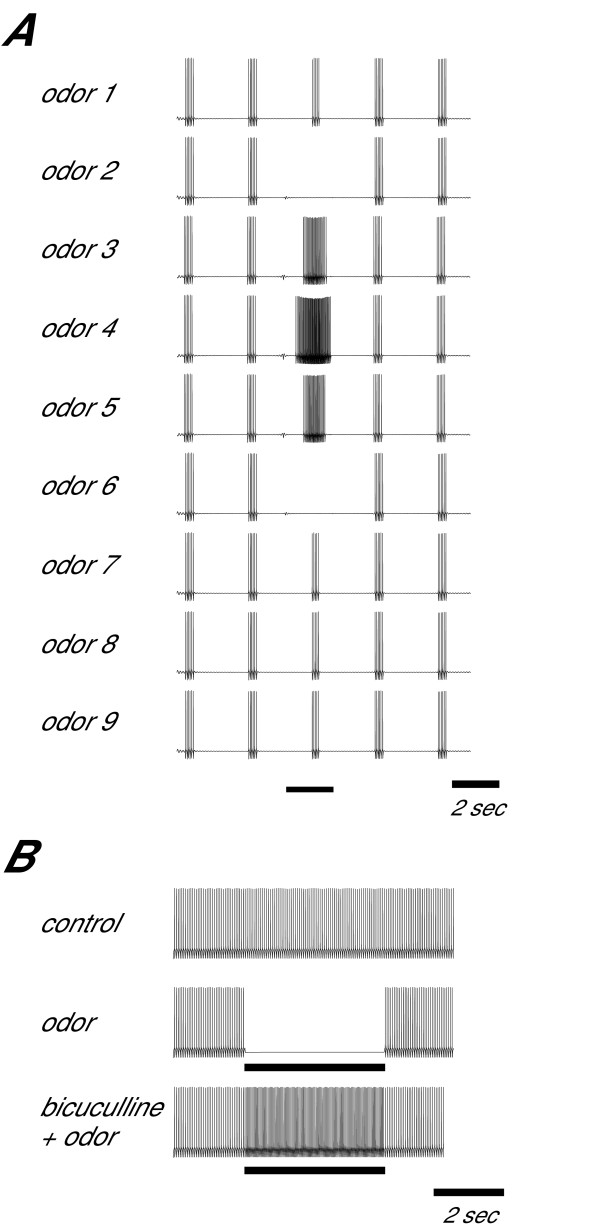
**Non-topographical contrast enhancement replicates the canonical observation of olfactory contrast enhancement. A. **A model mitral cell's response to the sequential presentation of a homologous series of nine different odorants. Nonspecific sinusoidal excitation was added to replicate the respiration-linked activity observed in the cell recorded by these authors (see Methods). The homologous odor series was simulated by altering the odor ligand-receptor affinity of the OSNs that project to the mitral cell depicted from near zero to a maximal value (at odor 4) and back along the trajectory of a normal distribution. The ligand-receptor affinities of the OSNs associated with the other nine glomeruli were sampled randomly from another normal distribution, as if they were exhibiting similar sensitivity profiles to unknown odor series. Odors were applied for 2 seconds (horizontal bar). NTCE fully replicated the Mexican hat contrast enhancement function observed in a mitral cell reported by Yokoi *et al. *([10]; their Figure 2A). **B. **Response dependence on GABA_A _receptors. Odor-evoked inhibition in mitral cells can be transformed into excitation when bulbar GABA_A _receptors are blocked [10]. In the present model, blockade of GABA-ergic synapses from periglomerular cells effected this reduction of inhibition, replicating the results of Yokoi *et al. *([10]; their Figure 5D). A constant background stimulation was applied in all cases to generate tonic spiking so that inhibition could be observed. *Control*, no odor stimulus was applied. *Odor*, a 4 second stimulus (using odor 6 from Figure 6A) was applied (horizontal bar), evoking an inhibitory response in the mitral cell. *Bicuculline + odor*, the same stimulus was applied after blocking all periglomerular synapses onto mitral cells. NTCE replicated the effects of bicuculline application as shown by Yokoi *et al. *[10], though the effect was mediated via periglomerular cells rather than granule cells as proposed by those authors.

The clearest demonstration of contrast enhancement in the olfactory bulb has been provided by Yokoi and colleagues [10]. Briefly, these authors showed that an individual mitral cell could respond to systematically varied odorant stimuli with a classical Mexican hat function: e.g., one featured mitral cell exhibited no response to a 3-carbon *n*-aldehyde, inhibition to a 4-carbon *n*-aldehyde, excitation to 5-, 6-, and 7-carbon *n*-aldehydes, inhibition to an 8-carbon *n*-aldehyde, and no response to 9-, 10-, or 11-carbon *n*-aldehydes. Furthermore, administration of the GABA_A _antagonist bicuculline appeared to broaden this Mexican hat response, transforming an inhibitory mitral cell odor response into an excitatory response. While the authors attributed this effect to the blockade of granule cell-mediated inhibition, it is also consistent with a blockade of GABAergic periglomerular cell-mediated inhibition and with NTCE. We modeled these authors' data by presenting a systematically varying odorant series to our ten-glomerulus model (see Methods). As NTCE is independent of specific lateral projections, the model did not have to be adjusted to perform contrast enhancement along the particular axes of variation used to model the homologous series of odorants. NTCE was able to replicate the contrast enhancement function within model mitral cells based on periglomerular inhibition alone (Figure 6A; compare with Figure 2A in reference [10]). Furthermore, blockade of this periglomerular inhibition transformed an inhibitory mitral cell odor response into an excitatory response (Figure 6B; compare with Figure 5D in reference [10]).

### Interpretations of existing data

Contrast enhancement among the representations of structurally similar odorants has been widely attributed to a process of reciprocal inhibition between mitral cells mediated via mutual dendrodendritic synapses with the dendrites of inhibitory granule cells [10,78-81], though lateral interactions via periglomerular neurons have also been proposed to mediate this function [2,82,83]. Lateral projections and their putative formation of an inhibitory surround have been both explicitly and implicitly presumed to reflect the two physical dimensions afforded by the columnar structure of the olfactory bulb such that physically neighboring mitral cells mutually inhibit one another more strongly than do more distantly neighboring cells [2,10,38,79,80,84,85], perhaps by analogy with the retina [86]. However, as explained above, physical proximity-based solutions such as decremental lateral inhibition cannot effectively mediate similarity-dependent computations such as contrast enhancement in a high-dimensional modality such as olfaction.

This problem has been specifically investigated in the analogous honeybee antennal lobe, using calcium imaging of odor-evoked glomerular activity among convergent OSNs and projection neurons (PNs; analogous to vertebrate mitral cells). Increasing odorant concentrations increased glomerular activation levels and recruited additional glomeruli, as in the vertebrate OB, while PNs tended to become more inhibited as odorant concentrations increased, particularly within reasonable concentration ranges [87]. Furthermore, in a systematic study of the transformation between OSN and PN odor-evoked activity patterns, PN activity patterns were significantly better fit by a model using a functionally-based pattern of inhibition (comparable to that generated by NTCE) than by a model employing nearest-neighbor lateral inhibition [11]. Indeed, the best fits based on nearest-neighbor inhibitory projections were similar to control fits based on random interglomerular projections.

Even within the vertebrate olfactory bulb, the hypothesis of proximity-based representations of odor similarity, while popular, is contraindicated in the literature. Mitral cells innervating neighboring glomeruli exhibit unrelated odorant response profiles [88], in contrast to mitral cells innervating the same glomerulus, which have very similar profiles [89]. Furthermore, as odor quality is gradually changed, using common, behaviorally validated models for the systematic variation of odor quality [10,27,90,91], the glomeruli recruited and lost from the gradually shifting odor representation are broadly distributed across the bulb rather than being consistently adjacent [42,45,71]. Quantitative measurements of response-correlated maps have revealed broad regions of odor-evoked inhibition among mitral cells that do not directly sample from strongly activated glomerular domains [92], a result consistent with numerous studies suggesting that a net inhibition is a common response of mitral cells to odor or olfactory nerve stimulation [54,63,72,74,75,89,92]. While this result alone does not exclude the possibility of (broad) center-surround inhibition, it is more consistent with a widespread, locally-induced, nontopographical pattern of inhibition such as that produced by NTCE. Indeed, in support of the latter hypothesis, strong odor-evoked inhibition of mitral cells has been clearly observed at locations far removed from the region(s) of peak activation evoked by that odor [92], and *in vivo *recordings coupled with calcium imaging data have suggested that action potential amplitudes and evoked calcium transients in mitral cell lateral dendrites do not decline in amplitude with increasing distance from the soma, but instead are propagated nondecrementally, suggesting an axon-like mechanism [93]. Both of these results argue against a decremental, topographically localized projection of lateral inhibition. Finally, while granule cell-mediated lateral inhibition can affect spike timing in mitral cells [94], these inhibitory synaptic inputs, widely distributed along mitral cell lateral dendrites, may not be capable of preventing spike initiation in mitral cells, particularly given that mitral cell spikes may be initiated in the primary dendrite and even potentially the glomerular tuft [52,62,95]. This implicates glomerular circuitry, rather than external plexiform layer circuitry, as the primary determinant of whether a given mitral cell will respond to a given odor input with action potentials or with inhibition. However, the long projections of mitral cell lateral dendrites and their dense interconnectivity with granule cell dendrites are likely to underlie a network of mutual inhibition among mitral cells approaching all-to-all connectivity, and hence are likely to be capable of performing secondary computations upon arbitrarily high-dimensional maps. The mitral-granule network is therefore potentially well suited to transform glomerular output in accordance with odor learning, presumably through the learned modification of mitral-granule reciprocal synapses and learning-correlated granule cell neurogenesis [96-99], and perhaps involving the generation of field oscillations and the synchronization of mitral cell spikes [94,100-103]. Indeed, a contrast enhancement transformation based on local field oscillations and spike synchronization has been modeled in antennal lobe PNs, analogous to mitral cells [76,104].

In the interests of simplicity, some known glomerular circuit elements were omitted from the present model. First, periglomerular cells deliver GABA_B_-ergic and D2 dopaminergic presynaptic inhibition onto the axon terminals of convergent OSNs [105-109]. This feedback inhibition regulates OSN glutamate release and has been proposed to help normalize OSN synaptic output with respect to concentration; such an effect would be expected to facilitate NTCE. Second, mutual excitation among mitral cells innervating the same glomerulus [110-113] would be expected to underlie cooperativity in the glomerular output response, sharpening the distinction between highly activated glomeruli in which mitral cells generate spikes and more modestly-activated glomeruli in which incoming OSN activity is dissipated without evoking mitral spikes. Third, self- (and potentially mutual) inhibition among PG cells, while hindering PG cell spike initiation, actually depolarizes PG cells and hence may have a potentiating effect on their graded inhibition of mitral cell apical dendrites [114]. This putative positive feedback loop may further contribute to the efficacy of PG inhibition of mitral cell spike initiation. Finally, some PG cells have axons that project to nearby glomeruli; while sparse, these projections have served as a basis for some models of lateral inhibition [82,83]. The utility of these projections is not clear; they potentially may serve to improve the functional differentiation of neighboring glomeruli with very similar molecular receptive ranges, such as might be generated via activity-dependent segregation [16].

The compartmentalized architecture of olfactory glomeruli may reflect a specific adaptation for the representation and processing of high-dimensional computational spaces within the physical constraints of two-dimensional cortical layers. If true, this would explain the observation that a functionally similar glomerular architecture has evolved independently several times in diverse phyla [115], and indeed is a nearly universal characteristic of complex olfactory systems. The limited diversity observed in these analogous olfactory structures (olfactory bulb, antennal lobe) may even suggest fundamental constraints on the effective processing of olfactory information above a certain level of complexity. This principle may also extend to other brain regions possessing glomerular architectures, notably the cerebellar cortex, implying that these circuits are also involved in the processing of high-dimensional representations.

## Conclusion

The NTCE algorithm presented here is the basis of a novel theory of bulbar function, integrating diverse data sets gathered by several laboratories. Fundamentally, it is a winner-take-most algorithm utilizing local feed-forward inhibition and global feedback inhibition to generate the competitive interactions among glomeruli typically associated with lateral inhibitory projections. Most importantly, it solves the problem of how to represent and process intrinsically high-dimensional sensory data within a physically two-dimensional neural cortex, while offering an explanation of the patchy, discontinuous odor quality maps observed in the olfactory bulb. As a model of contrast enhancement, it replicates the results of Yokoi *et al. *[10] illustrating the canonical Mexican hat function within single mitral cells and the dependence of this function on bulbar GABA_A _receptors. NTCE innately distributes inhibition among mitral cells according to the similarities in their molecular receptive ranges, an intractable problem for mechanisms based on lateral inhibitory projections embedded in discontinuous feature maps. Unlike mechanisms based on decremental lateral inhibitory projections, NTCE is independent of the physical proximity of similarly-tuned glomeruli, an important advantage given the broadly distributed representations of odor stimuli observed in the olfactory bulb. It offers a mechanism for the normalization of odorant representations with respect to concentration, reflecting the observation that mitral cell spike counts do not monotonically increase in proportion to primary sensory neuron spike counts as odorant concentrations are increased. Rather, it predicts complex effects on mitral cell activity patterns, including fast initial inhibition in response to odors and a variety of responses to odor concentration series owing in part to the unpredictable pattern of associations of multiple odor ligands with different odorant receptors. Finally, NTCE depends only on known properties of the olfactory bulb input layer, including its cellular morphologies and connectivity, passive membrane properties, active electrophysiological and pharmacological responses, and specialized glomerular architecture, to enable robust contrast enhancement in an objective, externally-defined, and high-dimensional similarity space.

## Methods

### Illustrative model of NTCE

NTCE depends upon two interacting computational mechanisms, each of which is consistent with available anatomical and physiological data. The first of these mechanisms, based on the rapid shunt inhibition of activated mitral cells by coactivated periglomerular spines, narrows the population of activated mitral cells with respect to the population of activated glomeruli such that only the most strongly activated subset of activated glomeruli generates action potentials in its associated mitral cells. Crucially, this contrast enhancement mechanism is independent of interglomerular projections, depending only upon relative degrees of glomerular activation and hence naturally inheriting the topology of odor similarity that is generated by the olfactory receptor complement. The second mechanism, essentially a global negative feedback loop, mitigates the effects of absolute odor concentration, thus enabling mitral cell activity patterns to reflect *relative *odor ligand-receptor affinities and efficacies and hence preserve the representation of odor quality across a reasonable concentration range.

The model depicted in Figure 3 was composed in Matlab (Mathworks, Natick, MA) and illustrates the first of these two mechanisms. For simplicity of illustration, odor concentrations are held constant. Mitral cells (Mi_in_) and periglomerular cells (PG) within a given glomerulus independently receive identical levels of direct sensory input (depending on the odor ligand-receptor affinity of their OSNs; *abscissa*), although they respond to this input differently based on their passive membrane properties (see Results and Discussion). As neuronal activation levels in this illustration (*ordinate*) depend upon ligand-receptor binding between the odorant and the olfactory receptor species projecting to the glomerulus in question, the direct activations of both Mi and PG cells vary sigmoidally as a function of odor ligand-receptor affinity:

{Miin,PG}=[K(1/1+(Y1/2A)m)]     (1)
 MathType@MTEF@5@5@+=feaafiart1ev1aaatCvAUfKttLearuWrP9MDH5MBPbIqV92AaeXatLxBI9gBaebbnrfifHhDYfgasaacH8akY=wiFfYdH8Gipec8Eeeu0xXdbba9frFj0=OqFfea0dXdd9vqai=hGuQ8kuc9pgc9s8qqaq=dirpe0xb9q8qiLsFr0=vr0=vr0dc8meaabaqaciaacaGaaeqabaqabeGadaaakeaacqGG7bWEcqqGnbqtcqqGPbqAdaWgaaWcbaGaeeyAaKMaeeOBa4gabeaakiabcYcaSiabbcfaqjabbEeahjabc2ha9jabg2da9iabcUfaBjabbUealjabcIcaOiabigdaXiabc+caViabigdaXiabgUcaRiabcIcaOmaalaaabaGaeeywaK1aaSbaaSqaaiabigdaXiabc+caViabikdaYaqabaaakeaacqqGbbqqaaGaeiykaKYaaWbaaSqabeaacqqGTbqBaaGccqGGPaqkcqGGDbqxcaWLjaGaaCzcamaabmaabaGaeGymaedacaGLOaGaayzkaaaaaa@4E88@

where *K *is a scaling constant determining relative excitability (due to input resistance), *Y*_1/2 _is the odor ligand-receptor affinity at which half-maximal output activity would be evoked in the mitral or PG cell via direct sensory input, *A *denotes the odor ligand-receptor affinity variable (*abscissa*), and *m *is the cooperativity (Hill equivalent; [116]) of each process. Net mitral cell output (Mi_out_) – i.e., the generation of spiking activity – depends on both direct mitral cell activation by sensory input (Mi_in_) and on the secondary inhibitory influence of periglomerular cell activity (PG), resulting in a net activation function for mitral cell output that depends on the difference between these two sigmoids:

Miout=[Ke(1/1+(Y1/2(e)A)me)]−[Ki(1/1+(Y1/2(i)A)mi)]     (2)
 MathType@MTEF@5@5@+=feaafiart1ev1aaatCvAUfKttLearuWrP9MDH5MBPbIqV92AaeXatLxBI9gBaebbnrfifHhDYfgasaacH8akY=wiFfYdH8Gipec8Eeeu0xXdbba9frFj0=OqFfea0dXdd9vqai=hGuQ8kuc9pgc9s8qqaq=dirpe0xb9q8qiLsFr0=vr0=vr0dc8meaabaqaciaacaGaaeqabaqabeGadaaakeaacqqGnbqtcqqGPbqAdaWgaaWcbaGaee4Ba8MaeeyDauNaeeiDaqhabeaakiabg2da9iabcUfaBjabbUealnaaBaaaleaacqqGLbqzaeqaaOGaeiikaGIaeGymaeJaei4la8IaeGymaeJaey4kaSIaeiikaGYaaSaaaeaacqqGzbqwdaWgaaWcbaGaeGymaeJaei4la8IaeGOmaiJaeiikaGIaeeyzauMaeiykaKcabeaaaOqaaiabbgeabbaacqGGPaqkdaahaaWcbeqaaiabb2gaTnaaBaaameaacqqGLbqzaeqaaaaakiabcMcaPiabc2faDjabgkHiTiabcUfaBjabbUealnaaBaaaleaacqqGPbqAaeqaaOGaeiikaGIaeGymaeJaei4la8IaeGymaeJaey4kaSIaeiikaGYaaSaaaeaacqqGzbqwdaWgaaWcbaGaeGymaeJaei4la8IaeGOmaiJaeiikaGIaeeyAaKMaeiykaKcabeaaaOqaaiabbgeabbaacqGGPaqkdaahaaWcbeqaaiabb2gaTnaaBaaameaacqqGPbqAaeqaaaaakiabcMcaPiabc2faDjaaxMaacaWLjaWaaeWaaeaacqaIYaGmaiaawIcacaGLPaaaaaa@689D@

in which subscript *e *denotes the parameters of mitral cell direct excitation (Mi_in_) and subscript *i *denotes the inhibitory effect of periglomerular cell activity (PG). In the example shown in Figure 3, *K*_*e *_= 1, *K*_*i *_= 0.6, *Y*_1/2*(e) *_= 10^-5 ^M, *Y*_1/2*(i) *_= 10^-4 ^M, and both *m *terms are unity.

Contrast enhancement is typically described with an on-center/inhibitory surround, or Mexican hat, function [117]; Figure 3, *inset*). Traditional Mexican hat functions in one dimension are typically modeled as a difference of Gaussians, or as the difference between two differences of sigmoids. Differences between sigmoids of equal maxima (K_e_, K_i_) and cooperativities (m_e_, m_i_) yield bell-like curves; the difference between two bell-like curves (whether gaussians or differences of sigmoids) generates a Mexican hat function given that the excitatory difference is of greater maximum magnitude and the inhibitory difference is of greater breadth. The corresponding function in NTCE is one half of a Mexican hat, modeled as a difference between sigmoids in which K_e _> K_i _and Y_1/2(e) _< Y_1/2(i) _and referred to as a *half-hat *function (Figure 3, *Mi*_*out*_; equation 2). Mexican hat functions of arbitrary dimensionality can be generated by rotating this half-hat function around the coordinate of its maximum activation level in a given context. When K_e _> K_i _and Y_1/2(e) _< Y_1/2(i) _– both conditions which are favored by the smaller volume and higher input resistance of periglomerular cell spines compared with mitral cell dendrites [118] – the output process Mi_out _(equation 2) exhibits contrast enhancement along the axis of odor ligand-receptor affinity *A *(see Results and Discussion).

### Compartmental model of NTCE

The compartmental model depicted in Figures 4 and 5 was composed in the neural simulation language NEURON [49-51]. Three cell types were explicitly modeled: mitral cells, periglomerular cells, and olfactory sensory neuron (OSN) axonal arbors. To minimize unconstrained parameters, the ET/SA network [2] was modeled as a set of delayed inhibitory synapses from each OSN to all mitral cells sampling from glomeruli other than the one to which that OSN projected, hence abstracting the projection of interglomerular inhibition mediated through the chain composed of local external tufted cells, short-axon cells, and periglomerular cells in other glomeruli. Granule cells and other post-glomerular circuitry were omitted. For simplicity, AMPA and NMDA glutamatergic synapses were combined into a single mechanism. All synapses were thresholded and graded so as to reflect either genuinely graded synaptic communication *in vivo *(e.g., PG to Mi synapses) or the spike density functions of large neuronal populations (e.g., convergent OSN inputs to PG and Mi cells), and approached steady-state exponentially.

#### Mitral cells

The mitral cell model was modified from that of Chen and colleagues [52,53], which is based on a simple complement of Hodgkin-Huxley currents (I_Na_, I_K_) with heterogeneous expression levels and optimized for its spike initiation properties. Model morphology and kinetic parameters were as in reference [53]. Specifically, two apical dendritic tufts fed into a single primary dendrite, which connected with a soma, an axon hillock, and two secondary dendrites, all of which expressed I_Na _at relatively low levels. The axon hillock in turn was connected to an initial segment and an axon with five node/internode pairs; the initial segment and nodes contained high levels of I_Na_, facilitating spike initiation. For the present simulations, glutamatergic excitatory and GABA_A_-ergic inhibitory synaptic inputs were inserted at the distal ends of apical dendrites.

#### Periglomerular cells

The periglomerular soma was connected to two narrow dendrites (20 um length, 1 um diam) from which protruded spine shafts and bodies (1 um diam). All periglomerular synaptic inputs and outputs were localized on spine bodies. While the axon contained only Hodgkin-Huxley currents, the dendrites and spines additionally contained a complement of currents capable of replicating low-threshold calcium bursts as observed in periglomerular cell recordings [55]; see also [58]. Each current mechanism was constructed in the NMODL language [119], and in some cases adapted from existing mechanisms as follows: mammalian hippocampal Hodgkin-Huxley sodium and potassium currents [120], low-threshold T-type calcium current [121], periglomerular cell hyperpolarization-activated (H-type) cation current [122], A-type inactivating potassium current [123], and a calcium diffusion model [120]. The kinetic parameters of each of these model mechanisms were retained, and have been made available on ModelDB [124] by their respective authors.

#### OSN axonal arbor

The OSN arbor was a passive, single-compartment model, serving as a source for graded glutamatergic excitation of mitral and periglomerular neurons; this graded excitation represented the spike density function of a large and highly convergent population of similarly tuned spiking neurons.

#### Uniglomerular model

The uniglomerular compartmental model included only mitral and periglomerular cells and OSN axonal arbors, and is directly comparable to the illustrative model described above and in Figure 3. OSN inputs excited both periglomerular cell spines and mitral cell distal dendrites via graded glutamatergic synapses, while periglomerular cell spines inhibited mitral cell distal dendrites via graded GABA_A_-ergic synapses (E_Glu _= 0 mV; E_GABA _= -80 mV). All synaptic time constants were set to 1 ms to reflect multiple, imprecisely timed inputs. The uniglomerular network was stimulated with four different levels of olfactory sensory neuronal input (12.6, 20, 90, or 140 pA injected into the OSN terminal); for each level of sensory input, the evoked activity was measured in (a) mitral cell somata in the absence of a PG-to-Mi inhibitory synapse, (b) mitral cells in the presence of a PG-to-Mi inhibitory synapse, and (c) periglomerular cells (Figure 4).

#### ET/SA network

Glomerular activation levels contribute to odor representations in that they reflect the pattern of relative affinities that the olfactory receptor complement has for odor ligands. However, ligands of lower affinity presented at higher concentrations can evoke levels of glomerular activation indiscriminable from those evoked by high-affinity ligands at lower concentrations [38]. This enables changing odor concentrations to degrade the effectiveness of contrast enhancement and the integrity of the olfactory representation. This problem can be resolved by normalizing glomerular output with respect to the sum of activity across all glomeruli [125], thereby dynamically regulating the stringency of the bulb's selectivity for odorant features [126]. In the olfactory bulb, this function is attributable to the ET/SA lateral excitatory network formed by external tufted and short-axon cells [2,3]. Short-axon cells (the term is a misnomer) are indirectly activated by sensory input (Figure 1), ramify broadly and isotropically within the glomerular layer for distances of up to 850 um (interconnecting areas separated by up to 30 glomerular radii), and form excitatory synapses upon distant periglomerular neurons, which in turn inhibit local mitral cells [2]. While the original study describing this ET/SA network proposed a center-surround topology [2], the observation that short-axon cells also form excitatory synapses upon one another and upon (excitatory) external tufted cells in multiple glomeruli, which in turn excite other SA cells, indicates the existence of a broad lateral excitatory network across the bulb. As short-axon cells are few in number and diverge broadly, sampling from 2–4 glomeruli and exhibiting extensive axonal branching throughout the glomerular layer, the ET/SA network is a wholly appropriate mediator of a broad and relatively indiscriminate projection of inhibition upon the mitral cell population. In the present model, ET/SA network activity fed back inhibition upon each mitral cell from all other glomeruli, hence translating the NTCE half-hat function on its abscissa in proportion to net bulbar input intensity such that only a small and consistent population of the most strongly activated mitral cells overcame this inhibition. In this way, NTCE could also establish a degree of concentration-independence in the secondary olfactory representation (i.e., mitral cell ensemble activity), in a manner consistent with experimental data and likely to facilitate the recognition of odor representations across concentrations. Signals reflecting absolute odor concentration could still be discerned from other response properties such as mitral cell spike latencies or tufted cell activity [77]; however, this question was not addressed in the present report.

#### Contrast enhancement reflected in individual mitral cells

In order to replicate Figure 2A from Yokoi *et al. *[10], the canonical demonstration of olfactory bulb contrast enhancement, the ten-glomerulus model was adapted in two ways. First, a 0.5 Hz sinusoidal input current was injected into the mitral cell distal glomerular tuft to replicate the artificial respiration-associated background activity depicted in that paper. Odors were then presented normally. Second, the sequential presentation of a homologous series of odorants was modeled as follows. All molecular receptive ranges were modeled as normal distributions of odor ligand-receptor affinities. As each subsequent odorant in the homologous series was presented, the affinity of the glomerulus of primary interest for that odorant first increased and then decreased along the trajectory of a normal distribution. The affinities of the other nine glomeruli for each odorant were also drawn from normal distributions, but randomly rather than sequentially, simulating a population of glomeruli with unknown tuning curves, the activity of which would vary unpredictably with changes in odor stimulation. Similar results were obtained when the affinities of the other nine glomeruli were simply held constant (data not shown). Data were high-pass filtered after generation to emphasize spiking activity and hence resemble extracellular recordings. Yokoi *et al. *[10] also illustrated that blockade of bulbar GABA_A _receptors with bicuculline could transform an mitral cell inhibitory odor response into a neutral or excitatory response. Using the ten-glomerulus model, we applied an odorant stimulus with moderate affinity for our glomerulus of interest, evoking an inhibitory response. We then blocked periglomerular synapses onto mitral cells and applied this stimulus again in order to replicate Figure 5D from Yokoi *et al. *[10].

## Authors' contributions

ThAC conceived and designed the study, coded and analyzed models in Matlab and NEURON, and drafted the manuscript. PS coded and analyzed models in NEURON.
